# 135. Impact of *Clostridioides difficile* Toxin Confirmation on Subsequent *C. difficile* Treatment in a Rural Health System (IT-CDI)

**DOI:** 10.1093/ofid/ofad500.208

**Published:** 2023-11-27

**Authors:** Jacquelyn Crawford, David B Cluck, Jennifer Tharp, Mathew Finniss, Paras Patel

**Affiliations:** Johnson City Medical Center Ballad Health, Johnson City, Tennessee; ETSU Bill Gatton College of Pharmacy, Johnson City, Tennessee; Ballad Health - Johnson City Medical Center, Johnson City, Tennessee; East Tennessee State University, Kingsport, Tennessee; Johnson City Medical Center, Johnson City, Tennessee

## Abstract

**Background:**

In January 2020, Ballad Health instituted the American College of Gastroenterology recommended algorithm for CDI diagnosis consisting of a reflex toxin confirmation post positive *C. difficile* PCR result. The study was designed to assess how the addition of the toxin confirmation impacted management of CDI in the inpatient setting.

**Methods:**

IT-CDI was a retrospective, multicenter, observational quasi-experimental study of 200 patients. Patients included in the study were > 17 years old with a positive *C. difficile* PCR and evaluated in two cohorts, pre-toxin confirmation versus post-toxin confirmation testing. The primary outcome was to determine if adding *C. difficile* toxin confirmation testing reduced antibiotic prescribing for the treatment of CDI. Secondary outcomes included duration of CDI treatment, impact of infectious diseases consultation, length of stay, accrued costs for *C*. *difficile* testing and treatment, and new incidence of vancomycin-resistant *Enterococcus* infection.

**Results:**

A significant reduction in initiation of treatment was found between cohort 1 (98%) versus cohort 2 (77%) although 54% of toxin-negative patients in cohort 2 still received CDI-targeted therapy. There were also significant reductions in duration of treatment from cohort 1 vs cohort 2 (median 13 vs 12; p=0.002) and hospital length of stay between toxin-positive patients versus toxin-negative patients in cohort 2 (median 13 vs 12; p=0.0027). Infectious disease consultation was not associated with a reduction in treatment and overall incidence of vancomycin-resistant *Enterococcus* infection was rare. Additionally, results demonstrated that toxin confirmation testing did not significantly reduce the total cost associated with testing and treatment which included the price of each lab test completed and the sum of inpatient CDI-targeted antimicrobial therapy per patient from cohort 1 vs cohort 2 (median $56 vs $57; p=0.3356).

**Conclusion:**

The addition of *C*. *difficile* toxin confirmation testing to hospital protocol reduced antibiotic prescribing for the treatment of CDI by 21%. However, over half of toxin-negative patients still received CDI antibiotic treatment. Toxin confirmation did not reduce length of stay nor total costs associated with testing and treatment.

**Disclosures:**

**David B. Cluck, PharmD, BCPS, BCIDP, AAHIVP**, Astellas: Advisor/Consultant

